# Detecting changes in help seeker conversations on a suicide prevention helpline during the COVID− 19 pandemic: in-depth analysis using encoder representations from transformers

**DOI:** 10.1186/s12889-022-12926-2

**Published:** 2022-03-18

**Authors:** Salim Salmi, Saskia Mérelle, Renske Gilissen, Rob van der Mei, Sandjai Bhulai

**Affiliations:** 1 Centrum Wiskunde & Informatica, Amsterdam, the Netherlands; 2 113 Suicide Prevention, Amsterdam, the Netherlands; 3 Vrije Universiteit Amsterdam, Amsterdam, the Netherlands

**Keywords:** Natural language processing, Suicide chat counseling, Machine learning, BERT

## Abstract

**Background:**

Preventatives measures to combat the spread of COVID− 19 have introduced social isolation, loneliness and financial stress. This study aims to identify whether the COVID-19 pandemic is related to changes in suicide-related problems for help seekers on a suicide prevention helpline.

**Methods:**

A retrospective cohort study was conducted using chat data from a suicide prevention helpline in the Netherlands. The natural language processing method BERTopic was used to detect common topics in messages from December 1, 2019 until June 1, 2020 (*N* = 8589). Relative topic occurrence was compared before and during the lock down starting on March 23, 2020. The observed changes in topic usage were likewise analyzed for male and female, younger and older help seekers and help seekers living alone.

**Results:**

The topic of the COVID-19 pandemic saw an 808% increase in relative occurrence after the lockdown. Furthermore, the results show that help seeker increased mention of thanking the counsellor (+ 15%), and male and young help seekers were grateful for the conversation (+ 45% and + 32% respectively). Coping methods such as watching TV (− 21%) or listening to music (− 15%) saw a decreased mention. Plans for suicide (− 9%) and plans for suicide at a specific location (− 15%) also saw a decreased mention. However, plans for suicide were mentioned more frequently by help seekers over 30 years old (+ 11%) or who live alone and (+ 52%). Furthermore, male help seekers talked about contact with emergency care (+ 43%) and panic and anxiety (+ 24%) more often. Negative emotions (+ 22%) and lack of self-confidence (+ 15%) were mentioned more often by help seekers under 30, and help seekers over 30 saw an increased mention of substance abuse (+ 9%).

**Conclusion:**

While mentions of distraction, social interaction and plans for suicide decreased, expressions of gratefulness for the helpline increased, highlighting the importance of contact to help seekers during the lockdown. Help seekers under 30, male or who live alone, showed changes that negatively related to suicidality and should be monitored closely.

## Background

The spread of the novel coronavirus (COVID-19) has put the lives in danger of many people around the world [1, 2]. Officially declared a pandemic, the measures to contain the spread caused many businesses to lose their income and the economy became uncertain. These problems emerged quickly after the virus took over. However, there is another, less obvious problem that the pandemic created.

For many people, the virus causes distress; for some, this can take the form of impaired mental health. In the months after the discovery of the virus on 27th December 2019, studies already found a decline in public mental health [3]. More importantly, the virus and the measures taken to contain it may affect risk of suicide [4, 5]. Scientists have expressed the fear that the number of suicides would rise, as government policy and measures to contain it would result in unemployment, financial stress, social isolation and loneliness [6]. O’Conner et al. observed an increase in suicidal ideation during the initial weeks of the lockdown in the UK [7]. Fortunately, the number of suicides has not increased in the first months of the pandamic [8, 9]. However, this does not provide a complete picture of COVID-19’s impact. We need to remain vigilant.

In what way does COVID-19 affect people with suicidal ideation? The virus introduced financial uncertainty, and social distancing became the new norm. People no longer had easy access to their social or professional outlets, education was disrupted, and there was an increase in bereavement and traumas. With a lack of social interaction, adolescents are particularly vulnerable because they are in an important period for social and emotional development [10, 11]. Did any of the pandemic’s changes contribute to a change in the problems that people with suicidal behavior or ideation experience?

The current study explores the impact of the COVID-19 pandemic in a specific at-risk population, i.e. help seekers that contact a suicide prevention helpline in the Netherlands (113 Suicide prevention). Suicide prevention helplines provide an accessible means of obtaining information on people who are struggling with suicidality. These helplines exist to counsel people through this struggle. These help seekers with suicidal ideation can contact the anonymous helpline by phone or chat. The chat data from these helplines can be used in combination with natural language-processing techniques to gain insights into the problems of people with suicidal thoughts during the pandemic.

Several studies have developed algorithms to detect changes in text content. Blei and Lafferty, as well as Wang et al. presented methods that model topic changes over time [12, 13]. In the domain of suicide prevention, Kumar et al. used topic modeling to detect changes in suicide-related content found in social media following celebrity suicides to measure their impact [14]. To the best of our knowledge, no study has been conducted to apply state-of-the-art NLP within the context of suicide prevention helplines.

With more than 100,000 conversations with help seekers in 2020, the chat service of the national suicide prevention helpline in the Netherlands presents an opportunity: these conversations can be analyzed during the COVID-19 pandemic in comparison with the conversations before. The potential changes can be observed from the logs of the chat service.

This study therefore aims to identify whether the COVID-19 pandemic is related to changes in suicide-related problems for help seekers on a suicide prevention helpline. A second aim is to detect changes in specific risk groups, e.g. male and young help seekers and help seekers alone. By doing so, the current study may inform suicide prevention strategies for organizations in public and mental health that help people with suicidal ideation and inform other national and international helplines about the application of this innovative methodology.

To this end, this paper intends to answer the following questions:

- What are the most common conversation topics of help seekers on the helpline?
- Has the occurrence of conversation topics changed after government measure for COVID-19 containment were introduced?
- Do different demographics show different changes in topic occurrence?

When it comes to different demographics and how they are affected, we hypothesize the following: The first hypothesis is that the observed changes in topic usage will differ between men and women, due to men being more sensitive to social-economic change [15]. Furthermore, we also suspect different outcomes for older and younger help seekers due to the difference in social economic impact the government measures. The second hypothesis is that the observed changes in topic usage will differ between people younger than 30 and older than 30, since adolescents particularly are at risk and even before the pandemic suicide rates were rising among young people in certain high-income countries [7, 16]. Finally, due to the social isolation of a lockdown, the third hypothesis is that people who live alone will show different topic usage from people who don’t live alone [17].

## Methods

Due to the nature of helpline chat data, we conducted a retrospective cohort study. To answer the first question, topics were extracted from the chats, using a unique topic modeling method tailored to conversational data. To answer the second and third questions, conversation topics were compared before and after the introduction of the COVID-19 measures.

### Sample

This study is centered around the first lockdown in the Netherlands, when the coronavirus first started to spread and impact the public. We believe that this period will show the greatest disparity between conversation topics. This lockdown took place on the March 23, 2020. Date spanned a roughly 6-month period, 3 months before and 3 months after the measures were announced (December 1, 2019–May 31, 2020). Conversations were used from December 1st onward to roughly match the number of conversations in both time periods and to include enough conversations from a period no information on COVID-19 was known.

The 6-month period yielded 8589 chat conversations that passed an initial triage stage. Situations where the help seeker was injured or could not continue the conversation in a safe environment were interrupted by the triage. These help seekers were subsequently referred to emergency services. These conversations were therefore excluded from this analysis. We used no further inclusion criteria.

For the purposes of this study, an anonymized database of this chat service was used. This introduced no research burden for the help seekers and therefore reduced the chance of selection bias. Of these conversations, 5179 (60%) and 3410 (40%) took place before and after March 23, respectively. Of the total sample, 1635 (19%) were male, 6576 (77%) were female, and 378 (4%) indicated to have a gender identity other than male or female. Furthermore, 6610 (80%) of the help seekers were under 30 years old, and 1662 (20%) were over 30 years old. Finally, 807 (9%) of the help seekers lived alone. There was a faulty age value in 300 conversations and these were declared missing values. The category ‘other’ for gender contained a small number of conversations. To avoid issues with the sample size of different topics this category was excluded from the analysis.

### Data preprocessing

Chat transcripts are often noisy and unstructured. Unlike regular texts, a chat transcript is a conversation between two people. This causes it to evolve organically, usually leading to a wide variety of topics in a single document. Furthermore, chat transcripts often include slang, shorthand, and spelling mistakes. Therefore, text needed to be preprocessed so that the information could be structured.

The Natural Language Tool Kit [18] filtered out non-descriptive words based on a common stop word list. For the purpose of limiting the use of slang and shorthand, and also to eliminate spelling mistakes, the text was narrowed down to the 2000 most frequent words in the entire corpus.

For our study, we were interested in the conversation topics of the help seeker. We also knew that helpline counselors had not changed their protocols since the arrival of COVID-19. Therefore, the messages of the counselors were excluded. Messages of help seekers that were under three words were also excluded, to limit basic messages like greetings.

### Analysis

The topic model method used in this study is a clustering of embeddings, based on the BERTopic method [19]. The embeddings were obtained using Sentence-BERT [20], a modification of the Bidirectional Encoder Representations from Transformers (BERT) network [21] to efficiently create short text embeddings. The full message was used for these embeddings. We used the pretrained multilingual BERT model “distiluse-base-multilingual-cased” to obtain the embeddings [22].

Using Uniform Manifold Approximation and Projection for dimensionality reduction, the embeddings are reduced from 300 to 5 dimensions [23]. Finally, the embeddings were clustered with the Hierarchical Density-based spatial clustering of applications with noise (HDBSCAN) algorithm [24]. HDBSCAN identifies and excludes noisy data points from the clusters, contributing to greater clarity of the topic model.

Separate embeddings were created for each message of the help seekers. Because the messages get clustered, each chat is a combination of clusters. This method mimics the approach of traditional topic modeling techniques like Latent Dirichlet Allocation (LDA), in which each document is modeled as a distribution of topics [25]. BERTopic was preferred due to a poor fit of LDA when using the entire chat as input. Furthermore, due to the shorter length of the individual chat messages, we found the BERTopic method to provide better results over LDA, while needing less parameter tuning. This method was also compared to several other topic modeling methods, such as the contextual topic model [26], and found to provide the best fit on the data.

The clustering of chat messages resulted in 81 clusters. Some of the clusters were comprised exclusively of identical messages. For example, messages like “I don’t know” were used by many help seekers and therefore identified as their own clusters. As these clusters did not contain any relevant information, they were excluded from the results. We identified 15 clusters with this characteristic, resulting in 66 clusters from which the topics were derived.

Finally, three methods were used to obtain definitions for each of the topics. First, the preprocessed message contents were grouped according to their assigned cluster. The TF-IDF metric was used on the grouped text to generate a list of the top five words for each topic [19]. Second, we inspected a handful of the anonymized messages associated with each topic for extra context. Third, two experienced counselors also observed the top five words along with the anonymized messages for each topic and provided their definitions. Based on this information, the final decision on the topic definitions was made by the first and second author. They solved uncertainties and discrepancies by checking several anonymized messages from the topics and discussed additional insights. The first author selected these messages based on how central to the cluster these messages were.

Because the number of conversations between the two time periods was different, a relative measure was used to determine the difference in the use of a topic before and after the introduction of the COVID-19 measures. Let N_1_ and N_2_ denote the total messages for the first and second time periods respectively. Given n^t^_1_ and n^t^_2_ as the number of messages belonging to topic t in each time period, the change in topic occurrence becomes:

$${r}^t=\frac{n_2^t}{N_2}/\frac{n_1^t}{N_1}-1$$

Ultimately, this resulted in a rate of relative change for each topic, presented as a percentage in the results. These same methods were also used to compute the change in topic use for each of the different subgroups. To obtain the change for each topic among men, women, younger, older, and help seekers living alone, only the conversations that fell into that subgroup were included.

## Results

### Most important topics

Through the topic modeling of all 8589 chat conversations 66 topics were determined. The definitions as well as the frequencies and relative change of these topics can be found in Table 1. The most frequent topics concerned events the help seeker experienced or treatment the help seeker received in the previous days or weeks. For example, help seekers talked about treatments they received or if they had discussed their suicidal thoughts with a specialist.

**Table 1:** The frequency and definitions of topics

Frequency	Relative Change	Topic
6138	-1%	Description of events and treatment in recent days or weeks
5962	1%	Need to talk, difficulty talking to those around them
5076	-1%	Family circumstances
4984	3%	How the help seeker feels
4961	−3%	The help seeker doesn’t know what to do
4598	−1%	Thoughts and thought patterns
4511	−1%	Help seeker is seeking help / help isn’t working
4111	−1%	Life is pointless
3638	0%	Friendship
3476	−6%	Suicidal thoughts
2832	−2%	Desire to go to sleep or difficulty sleeping
2450	−5%	Help seeker, housemates, family are not at home
2439	−2%	Depression and stress
2288	5%	Psychologist or psychiatrist
2226	8%	Help seeker finds something difficult or awkward
2212	−1%	Help seeker has been trying things for a long time
1832	3%	Pain and sadness
1791	−2%	Problems with medication and alcohol
1732	−20%	School and study
1706	−9%	Help seeker is looking for a solution or can’t see a solution for their problems
1679	5%	Help seeker’s hopes and expectations
1589	−9%	Has plans die by suicide
1503	10%	Thanking the counselor
1463	−8%	Description of events and behaviors in the last month(s)
1305	1%	The help seeker has a lot on their mind and wants peace
1229	0%	Not being understood or not understanding others
1192	4%	No confidence in self or others
1182	−2%	Self-harm
1173	4%	Desire to be happy
1161	13%	Writing down thoughts, writing goodbye letters, and distraction by reading books
1142	5%	Question for the counselor
1139	4%	Panic and anxiety
1016	9%	Apologizing to counselor
970	11%	Desire to contact the helpline or someone else
887	−15%	Distraction by listening to music or reading
828	5%	Negative feelings or self-image and a desire for more positive thoughts
750	−21%	Distraction by watching TV, series or films
683	−2%	Crisis service
623	−14%	Planning to die by suicide at a specific location
620	2%	Help seeker can’t go on, has given up
608	6%	Children
606	6%	Help seeker finds it difficult to talk about it
583	5%	The help seeker has no more options apart from dying by suicide
567	808%	Coronavirus
549	4%	The help seeker asks how he/she can carry on living
500	12%	The help seeker is in his/her room
479	−8%	The help seeker has doubts or is uncertain
472	1%	Help seeker has worries about the future
462	−9%	Help seeker wants to tell their story
459	−1%	The help seeker is doing better
427	−5%	The help seeker is bleeding
417	−7%	Help seeker has no more energy
412	13%	Help seeker indicates that a proposed solution doesn’t work
412	−1%	Negative emotions and weeping
390	−5%	Feelings of guilt and responsibility
368	15%	Help seeker is grateful for being listened to
368	−9%	Means for suicide in the kitchen
358	3%	Help seeker is grateful for the conversation
337	16%	The help seeker is experiencing something for the first time
322	1%	The help seeker is safe
319	31%	Help seeker is at home
308	1%	Distraction by taking the dog for a walk
301	5%	Loss of control
273	−32%	Romantic relationships
271	18%	Involvement of police in the event of a suicide attempt or unsafe home situation
251	11%	Problems due to autism or ADHD

### Changes in topics occurrence

As expected, during the initial months of the pandemic, help seekers more often contacted from home and more often mentioned encounters with new situations. Conversations had more mentions of negative emotions. However, we also saw an increase in conversations that mentioned encounters with law enforcement, connected to suicide attempts or unsafe environments at home. Furthermore, problems with autism and ADHD appeared more often in chat conversations during the lockdown.

### COVID-19 as a conversation topic

The most extreme result was, perhaps unsurprisingly, the topic of the COVID-19 pandemic, which saw an 808% increase in relative occurrence after the introduction of COVID-19 containment measures. Table 2 shows some of the common problems help seekers mentioned, as well as some example chat messages related to these problems.

**Table 2:** Main categories within corona topic

COVID-19 related problem	Example chat message
Loneliness	The loneliness has become worse because of corona.
No distractions / lack of structure	I also don’t have a lot of distraction now because everything is closed due to corona.
Change in regular care	It seems to get more difficult in this corona period because ...
Feeling of getting locked up	… a fear getting stuck at home because of corona.
Fear of the virus	… with my fear of germs this whole corona situation is a nightmare come true.
Difficult situation at home	… and because of corona I work from home, it’s so stressful, and also not the best with my family.
(Threat of) unemployment	I was fired from my other job because of corona.
Increase of fear and depression	Due to this whole corona situation my anxiety is flaring up.
Concerns about somebody else	And I’m not allowed to go near my grandparents because my granddad has […] and if he gets corona he will not survive
Alcohol and drug abuse	With corona it’s worse than usual. I’m constantly worrying and then I drink […]

### Changes for gender, age, and living alone

Because relatively more women and young people contacted the chat service, their results are largely similar to the total results. Tables 3 and 4 show the most remarkable differences between the two gender and age demographics, respectively. Table 5 shows the biggest changes for help seekers who live alone.

**Table 3:** Comparison of topic change between men and women

Total	Women	Men	Topic
31%	44%	−15%	Help seeker is at home
12%	21%	−13%	The help seeker is in his/her room
6%	5%	−14%	Help seeker finds it difficult to talk about
4%	2%	24%	Panic and anxiety
4%	13%	−21%	Desire to be happy
3%	− 2%	43%	Help seeker is grateful for the conversation
1%	5%	−26%	The help seeker has a lot on their mind and wants peace
−2%	−5%	43%	Crisis service
−5%	2%	−29%	Feelings of guilt and responsibility
−7%	−10%	11%	Help seeker has no more energy

**Table 4:** Comparison of topic change between help seekers younger and older than 30

Total	Age < 30	Age > = 30	Topic
15%	31%	−12%	Help seeker is grateful for being listened to
13%	22%	−12%	Negative emotions and weeping
4%	15%	−21%	No confidence in self or others
4%	13%	−27%	Desire to be happy
3%	15%	−20%	Help seeker is grateful for the conversation
−2%	−5%	9%	Problems with medication and alcohol
−9%	−12%	11%	Means for suicide in the kitchen

**Table 5:** Topic change of help seekers who live alone

Total	Living alone	Topic
−20%	15%	School and study
−9%	52%	Has plans to die by suicide
−6%	27%	Suicidal thoughts
−2%	−41%	Self-harm
6%	−62%	Help seeker finds something difficult or awkward
1%	−40%	Help seeker is safe
−7%	70%	Help seeker has no more energy

After the introduction of COVID-19 measures, conversations with male help seekers more often involved feelings of panic, fear, and emergency care. They also expressed their gratitude more frequently.

When looking at changes between the different age groups after the introduction of COVID-19 measures, we saw that younger help seekers focused more on negative emotions. They also expressed a more frequent lack of confidence in themselves or others. Furthermore, the younger help seekers did more often express thankfulness for the conversation during the lockdown. On the other hand, older help seekers more often discussed problems with medication, alcohol, and methods of self-harm.

Help seekers that lived alone showed the most extreme changes between the two time periods. This is in part because this group was relatively small compared to the total number of help seekers. Nevertheless, there were striking changes in this group. Help seekers that lived alone more often talked about suicidal thoughts or plans, and also about having no energy. On the other hand, the conversations less often mentioned difficulty talking about their situation or about self-harm.

To summarize, there was an increase in topics indicating gratefulness for the helpline. Furthermore, there was a decrease in mention of plans for suicide and suicidal thoughts. Help seekers who live alone however, showed an increase on plans for suicide and suicidal thoughts. Male help seekers showed increase mention of panic and anxiety. Help seekers under 30 showed an increased mention of negative emotion and lack of self-confidence, while help seekers over 30 showed an increased mention of problems with medication or alcohol.

## Discussion

This study was conducted to inform suicide prevention strategies for organizations in public and mental health that help people with suicidal ideation. This was also the first study in the field of suicide prevention that used BERT embeddings resulting in an in-depth analysis of a large number of conversations using topic modeling. This study differs from previous studies on mental health conversational data. Examples of such studies dealt with real-time topic modeling of crisis chat helplines with LDA that leveraged experts during the training to improve the model [27], and modeling of a set amount of conversation stages [28].

The topic model found multiple changes in topic frequency in the overall help seeker population. Several of these changes can be explained by the government measure. Examples of these are that help seekers called more often from home, shared less about romantic relationships, and talked less about their education. These changes are in line with the government measures causing schools to close and recommendations to stay at home to keep social interaction to a minimum.

Certain changes pertaining to the functioning of the helpline were of interest. There was increased mention of thankfulness on the part of the help seekers for the conversation during the lockdown, indicating a desire for contact. This is an important finding since loneliness and thwarted belongingness are strongly associated with suicidal ideation and behavior [29]. In a study on reporting the COVID-19 related problems mentioned by help seekers, van der Burgt et al. found interruption of regular care and loneliness to be the most frequently mentioned [30]. Helplines can provide support in times of loneliness that has been amplified by the lockdown.

There was an increase in mentions of negative emotions and lack of self-confidence in chats, especially among younger help seekers. The mental health of this group was tracked in the UK by Pierce et al. [31]. They found an increase in mental distress in the general population during the pandemic and specific groups, like adolescents, were affected more significantly. A study on the age group 8–18 found indication that internalizing problems of this group increased during the pandemic [32]. We know that this group is already at risk for calling a suicide prevention help line. As an important period in their life for social emotional development and with government measures still persisting it is important to provide and promote means for this group to socially interact on a regular basis to improve their.

There was a decrease in mentions of specific plans and methods for suicide. Furthermore, helplines saw an initial decrease in usage of the helpline and the observed number of suicides during the early months of COVID-19, and there was no significant increase [9]. However, financial uncertainty and increased risk factors due to social isolation would expect the opposite. Suicide remains a complex phenomenon and we cannot yet explain how these findings relate.

There was also an increase in mentions of panic and anxiety among male help seekers. This could be because of the image of social economic change [15].

Among help seekers who live alone, an increase in mentions of specific suicide plans was observed. This group of people were hit harder by the pandemic than most help seekers, and this was apparent in the discussed topics. A possible contribution could be the decrease of their daily social contact during a lockdown. Combined with no contact at home, it could lead to more time with their own thoughts. The ruminative process affects the transition of defeat leading to entrapment which can then turn into suicidal ideation according to the model of O’conner et al. [29]. It will be important for policy makers to keep a close eye on how their actions affects people who live alone and provide leniency where possible so that contact for this at-risk group could still be possible.

### Limitations

While technology has progressed rapidly in recent years, natural language processing remains a randomized process. Outliers or “noise” in the data is therefore a factor to be aware of, and we acknowledge this as a limitation of this study. With this method, noise could manifest in three ways. First, the topic model does not classify every message. This feature has the benefit of reducing false positives within the topic model, based on the closeness to a cluster and the cluster size. However, this does also mean that some of the messages that could be considered relevant to the topic can fall outside the cluster. The second limitation stems from the cluster size. Topics that have a smaller size are more prone to fluctuation and therefore have lower statistical power. This effect is amplified when considering a smaller demographic, like male help seekers or help seekers who live alone. The third limitation concerns the accuracy of the BERT embeddings. While no model is perfect, the potential mismatch between the text data used for training the BERT model and the chat data is something to be aware of. The BERT model was trained on a concatenation of Wikipedia pages [22]. As our dataset includes mostly conversational data, there might be language usage specific to conversations that is therefore unseen by the BERT model. This could influence the number of messages classified as noise or wrongly assigned to a cluster.

### Implications

Several implications for counsellors in a suicide prevention helpline are recommended. Some of the key pillars that counsellors were trained to look for in a conversation were short-term coping, long-term hope to live, reasons to live, and decreasing accessibility of the means for suicide. Counsellors can direct the conversation to cover topics that are less often mentioned during the lockdown. For example, two of the three topics about short-term coping styles are less frequently reported by help seekers. Difficulty of finding distraction also appeared as an recurring topic in a study by van der Burgt et al. [30].

People who live alone have no household to infect and we recommend policy makers to treat this group accordingly, by allowing exceptions for those who need it, to work from the office for example. Negative emotions among young people could have an increased chance to go unnoticed due to the decreased social interaction. Allowing for social interaction outdoors, such as sporting events could be a possible way for younger people to be able to talk to others and express emotions they cannot at home. This is also important because it is part of forming their identity through interaction with peers [10, 11], which would also promote self-confidence.

### Future research

There are three main avenues of future work. First, the current method can be applied to other time periods. As of the time of writing, there has already been a second and third wave of the virus, and a second, stricter lockdown has gone into effect in the Netherlands. With distinctive periods, characterized by the different stages of lockdown, we can infer a better picture of help seeker trends over time. Second, we advise organizations that provide support through online counseling that are interested in obtaining insights on their demographic to consider this method. Third, topic interpretability can be improved. One approach involves levering the attention mechanism underlying the BERT model. A summary could potentially add semantic context that a list of important words could not. One potential way to do this is with transformer-based models for summarization [33, 34].

## Conclusion

This study offers new insights into a specific at-risk population for suicide by examining common problems that help seekers from a suicide prevention helpline experience. Although the number of suicides fortunately has not increased during the COVID-19 pandemic in the Netherlands in 2020, changes in frequencies of these problems during the lockdown were detected. Help seekers showed a decreased mention of topics involving distractions and plans or methods for suicide. Topics involving gratefulness for the conversation saw an increase in mention.

Furthermore, the model detected several changes in sub demographics of the help seekers. Among help seekers who live alone there was in increase in mention of plans for suicide. Male help seekers showed an increase mention of panic and anxiety. Help seekers under 30 showed an increase in mentions of negative emotions and lack of self-confidence.

It remains important, during this period of reduced social interaction, increased fear and uncertainty, to keep monitoring the changes over time, to allow for exceptions for younger, male or help seekers who live alone where possible, and to keep help and communication available.

## Data Availability

The datasets used and/or analysed during the current study are available from the corresponding author on reasonable request.

## Abbreviations

HDBSCAN: Hierarchical Density-based spatial clustering of applications with noise
LDA: Latent Dirichlet Allocation
BERT: Bidirectional Encoder Representations from Transformers
